# Application and Validation of Activity Monitors’ Epoch Lengths and Placement Sites for Physical Activity Assessment in Exergaming

**DOI:** 10.3390/jcm7090268

**Published:** 2018-09-11

**Authors:** Jungyun Hwang, Austin Michael Fernandez, Amy Shirong Lu

**Affiliations:** 1 Department of Communication Studies, College of Arts, Media and Design, Northeastern University, Boston, MA 02115, USA; fernandez.au@husky.neu.edu (A.M.F.); a.lu@northeastern.edu (A.S.L.); 2 Department of Health Sciences, Bouvé College of Health Sciences, Northeastern University, Boston, MA 02115, USA

**Keywords:** active video game, accelerometry, physical activity assessment, epoch, placement site, heart rate

## Abstract

We assessed the agreement of two ActiGraph activity monitors (wGT3X vs. GT9X) placed at the hip and the wrist and determined an appropriate epoch length for physical activity levels in an exergaming setting. Forty-seven young adults played a 30-min exergame while wearing wGT3X and GT9X on both hip and wrist placement sites and a heart rate sensor below the chest. Intraclass correlation coefficient indicated that intermonitor agreement in steps and activity counts was excellent on the hip and good on the wrist. Bland-Altman plots indicated good intermonitor agreement in the steps and activity counts on both placement sites but a significant intermonitor difference was detected in steps on the wrist. Time spent in sedentary and physical activity intensity levels varied across six epoch lengths and depended on the placement sites, whereas time spent from a 1-s epoch of the hip-worn monitors most accurately matched the relative exercise intensity by heart rate. Hip placement site was associated with better step-counting accuracy for both activity monitors and more valid estimation of physical activity levels. A 1-s epoch was the most appropriate epoch length to detect short bursts of intense physical activity and may be the best choice for data processing and analysis in exergaming studies examining intermittent physical activities.

## 1. Introduction

An accelerometer is an electromechanical device used to measure acceleration forces and thereby detect motions [1]. Since accelerometry functions are applicable to wearable activity monitors, accelerometer-based activity monitors have been widely accepted as a useful and practical device for monitoring and tracking physical activity as well as predicting energy expenditure [2]. Further, the use of accelerometer-based activity monitors significantly contributes to the field of physical activity and health, such as the development of physical activity classification [3,4], estimation of the mortality [5], and application for different research settings [6,7]. As such, physical activity assessment must be accurate; thus, researchers have validated accelerometer properties, placements, and/or data processing in regular physical activity settings [2] but seldom in exergaming settings.

Exergaming combines body movements and video gaming and requires bilateral coordination skills of both upper and lower limb movement for different movement patterns (e.g., punching, kicking, jumping) in response to visual cues [8]. Since exergaming increases energy expenditure and achieves moderate-to-vigorous levels of physical activity [9,10], it has been widely implemented in clinical settings [11] as well as in laboratory, home, schools, and the community [12] as an innovative and alternative strategy to promote physical activity and health. To our knowledge, no exergaming studies have processed accelerometry data into quantifiable and interpretable information involving different monitors, placement sites, epoch lengths, or activity cut-points [2]. There is thus an urgent need to validate the use of accelerometry for the assessment of physical activity in exergaming research.

In comparing subjective methods (e.g., diaries, questionnaires) for physical activity assessment, accelerometer-based activity monitors are regarded as the gold standard in detecting steps and quantifying the volume and intensity of physical activity [1]. Such activity monitors have been used in a wide range of applications and in a variety of clinical and research settings [2]. Despite their frequency of usage, validation studies have reported discrepancies in steps or physical activity levels when comparing activity monitors of different brands (e.g., activPAL, Hookie AM20, Polar Active vs. ActiGraph) at different placement sites [7,13,14]; these validation studies have mostly assessed regular physical activities (e.g., walking, running) [15,16] or free-living activities [15,17], but one recent study compared the output of different monitors (pedometer vs. accelerometer) in an exergaming setting [18].

One of the most commonly used activity monitor brands in physical activity research, ActiGraph has developed multiple generations of activity monitors [19]. Researchers have validated different ActiGraph activity monitors—including GT3X vs. GT1M [4], GT1M, GT3X, vs. GT3X+ [20], and recently, GT3X+ vs. GT9X [21]—placed at different sites such as hip vs. wrist [15,22,23] during various physical activities. Although a hip placement site has been validated as an ideal location for accurately measuring steps and physical activity level in regular physical activities [15], the evaluation of multiple placement sites (hip vs. wrist) in exergaming research is needed as more upper limb movements (unlike most regular physical activity) are required for exergaming [8]. In addition, validation studies mainly focusing on young people (from preschoolers to adolescents) have evaluated epoch lengths using different sets of activity cut-points [24,25,26], which impact the assessment of sedentary behavior and the different levels of physical activity intensity [25,27]. Since the exergaming play we chose to evaluate here features acute bouts of intermittent and spontaneous physical activity, shorter epochs might be a better choice for capturing short bouts of frequently occurring activity [2]. To date, there have been no studies comparing the effect of placement sites and epoch lengths on output especially from exergaming play or in young adults [2]; thus, the most appropriate accelerometer data collection and scoring protocol remains unclear.

Of particular relevance here, studies comparing physical activity levels from different epoch lengths have not validated theses assessments with absolute measures of exercise intensity via indirect calorimetry (e.g., oxygen uptake, metabolic equivalent) or relative measures of exercise intensity via heart rate (HR) monitoring (e.g., %HRmax, %HR reserve (HRR)) [25,26,28,29], which can be used as comparators to determine an appropriate epoch length for the accuracy of physical activity assessment. Whereas either relative or absolute measures can be used for classifying different levels of physical activity intensity [30], the use of relative measures in comparing epoch lengths should be more feasible and effective for such an assessment [4,31]. We believe that studies comparing epochs between activity counts and HR have never been reported, especially in an exergaming setting.

We aimed to examine the agreement of two recent generations of ActiGraph monitors (wGT3X-BT and GT9X Link, referred to below as wGT3X and GT9X, respectively) placed at different sites (hip and wrist). We sought to determine the most appropriate epoch length for physical activity assessment when validated using measurements of relative exercise intensity such as HR in healthy young adults in an exergaming setting. Our findings provide insight into effective data collection strategies for exergaming research, thereby improving the accuracy of physical activity assessment.

## 2. Materials and Methods

### 2.1. Participants

We recruited 47 healthy young adults of different ethnic backgrounds and both genders who spoke English from a university in the northeastern region of the United States via web advertisements and flyers. Participants were eligible if they met the following conditions: (1) were between 18 and 25 years old; (2) were free from physical disability (e.g., gait abnormalities); and (3) were not a current or former user of tobacco. Our study was approved by the Institutional Review Board of Northeastern University and all participants signed a written consent form for their participation.

### 2.2. Study Procedures and Instruments

We used a cross-sectional design and collected data from 22 March 2017 to 21 September 2017. Once a participant arrived at the laboratory, we provided the participant with an orientation on study procedures and potential risks. We measured their weight and height and computed their body mass index (BMI; kg/m^2^). We used ActiGraph tri-axial monitors (ActiGraph LLC, Pensacola, FL, USA) including wGT3X (46 × 33 × 15 mm, mass 19 g) and GT9X (35 × 35 × 10 mm, mass 14 g). We rotated and counterbalanced the placement of the ActiGraph monitors to avoid any potential order or placement effects.

Using the ActiLife software v.6.13.2 (ActiGraph LLC, Pensacola, FL, USA), we initialized four ActiGraph tri-axial monitors at a sampling rate of 30 Hz and set the Bluetooth wireless function for a wrist-worn GT9X to integrate with a Polar H7 Bluetooth heart rate sensor (Polar Electro Inc., Lake Success, NY, USA) for continuous heart rate measurement. We positioned a wGT3X with a belt clip and GT9X with an elastic belt at the anterior axillary line of the nondominant hip and another wGT3X with a nylon band and GT9X with a silicone band on the nondominant wrist [14]. We also placed a Polar H7 Bluetooth heart rate sensor on the chest with a soft textile strap.

After we confirmed that all devices worked properly, participants played for three 10-min segments for a total of 30 min. Each segment comprised 2 min of passive rest (standing) followed by 8 min of playing Kung-Fu for Kinect (http://www.kungfuforkinect.com), which involves upper and lower movements via a Kinect sensor on an Xbox One console (Microsoft Inc., Redmond, WA, USA). While playing the exergame, a participant could see his/her own body on the screen and fought enemies using his/her own moves in a 2D fighting adventure environment. When different enemies appeared on the screen, a participant engaged them with a variety of intermittent and spontaneous movement patterns and skills (e.g., jumping, punching, kicking). The intensity level of the exergaming was determined by continuous HR measurement as described above and self-assessment using the Borg rating of perceived exertion (RPE) [32] before and immediately after the 30-min exergaming play. We monitored the play time and recorded the start and end time of each interval on a study checklist.

### 2.3. Accelerometry Procedure and Data Reduction

The ActiGraph tri-axial monitors measure accelerations from the subject’s amplitude (g) and frequency (Hz) of movement in three individual axes (X axis: anterior-posterior, Y axis: vertical, Z axis: medial-lateral). Using the ActiLife software v.6.13.2, we transferred the collected data from the monitors and downloaded the activity counts from the three axes and vector magnitude (VM) obtained from all three axes (x^2^ + y^2^ + z^2^)^1/2^ with six epoch lengths (1, 5, 10, 15, 30, and 60 s). We used the two popular and validated Sasaki and Troiano’s activity cut-point sets [3,4] as appropriate for adults to estimate the amount of time spent in sedentary behavior (SB) and light (LPA), moderate (MPA), vigorous (VPA), and very vigorous physical activity (VVPA): (1) Sasaki’s cut-points [4] (e.g., a 60 s epoch (counts per minute, CPM): ≤150, 151–2690, 2691–6166, 6167–9642, and >9642) for the categories of SB, LPA, MPA, VPA, and VVPA, respectively, and (2) Troiano’s cut-points [3] (e.g., a 60 s epoch (CPM): ≤100, 101–2019, 2020–5998, and ≥5998) for SB, LPA, MPA, and VPA. We combined Sasaki’s VVPA with VPA for subsequent data analysis. Sasaki did not report classifications of either SB or LPA; thus, we used ≤150 and 151–2690 to define these categories, respectively, as 150 CPM may be the most appropriate cut-point to use to define SB for ActiGraph monitors [2,33]. We then converted the dataset into another of five shorter epoch lengths (1, 5, 10, 15, and 30 s) and recalculated the sedentary and physical activity intensity levels for subsequent data analysis. We also summed the step counts calculated from the built-in algorithm of the ActiLife software using a zero-crossing method based on raw accelerations from the vertical axis [1].

We downloaded HR data recorded at every second as a 10-s interval dataset. To compare intensity assessed indirectly via HR with the categories from activity counts in six epoch lengths, we calculated the amount of time spent in SB (<57), LPA (57–63), MPA (64–76), and VPA (>77) based on the categories of relative exercise intensity (%HRmax) [30] after adjusting for age-predicted maximal HR [208 − (0.7 × age)] [34].

### 2.4. Statistical Data Analyses

We ran statistical data analysis separately for the hip and the wrist. Of the 47 subjects, we analyzed 47 datasets from the wrist but only 45 datasets from the hip for activity counts due to a technical problem with two of the monitors. Additionally, of the 47 subjects, 41 datasets for heart rate were analyzed; five were excluded due to inappropriate data for analysis (namely, logging more than 25 of 30 min at the sedentary level) and a technical problem for one additional monitor.

We used intraclass correlation coefficients (ICC) to examine intermonitor agreement in steps and activity counts using the following categories [35]: poor (<0.5); moderate (0.5–0.75); good (0.75–0.9); and excellent (>0.9). We confirmed this using a Bland-Altman analysis to assess the mean bias and limits of agreement and calculated mean bias % as (GT9X − wGT3X)/mean% [36]. For the Bland-Altman plots and ICC analyses, we used Sasaki’s activity cut-points to compare steps, each axis count (CPM), and VM (CPM) between wGT3X and GT9X placed on the hip or the wrist. Additionally, due to the orientation difference of the wGT3X and GT9X monitors when worn on the wrist, we compared data from the Y and X axes in the GT9X to the data from the X and Y axes in the wGT3X, respectively, according to the manufacturer’s suggestion (J. MacDonald, written communication, May 2018) (see Supplemental Figure S1). We also performed a repeated measures ANOVA: (1) to assess the mean differences of steps, the three axes’ activity counts, and the VM between the monitors; (2) to test for an interaction for time spent in SB and the different levels of physical activity intensity in six epoch lengths with two monitors and two activity cut-point sets; and (3) to compare the mean amount of time spent in SB and different levels of physical activity intensity assessed via HR and activity counts (categorized separately using the two activity cut-point sets) in six epoch lengths averaged from two monitors. When a significant interaction was observed, we performed a Tukey’s post hoc test to identify pairwise differences. All statistical data analyses were conducted with IBM SPSS 24.0 (IBM Corp., Armonk, NY, USA). The criterion for significance was *p* < 0.05. Data are presented as mean ± standard deviation.

## 3. Results

### 3.1. Descriptive Analysis

Participants (*N* = 47; 25 males) were, on average, 21.4 ± 2.2 years old, 171.9 ± 10.6 cm in height, and 68.0 ± 16.6 kg in weight, and had a BMI of 23.2 ± 4.7 for body mass index. They consisted of 40.4% Caucasian, 2.1% African American, 44.7% Asian, 6.4% Hispanic/Latino, and 6.4% mixed races or ethnicities. All participants engaged in an approximately 30-min exergaming session. The mean HR during the exergaming was 130.1 ± 22.4 beats/min and the means of RPE before and after the exergaming were 8.3 ± 2.0 and 13.1 ± 2.8, respectively, indicating a moderate intensity level of physical activity.

### 3.2. Agreement between GT9X and wGT3X Placed on Hip and Wrist

As shown in Table 1, the ICC estimate with a 95% confidence interval indicated excellent agreement in steps, X axis, Y axis, and VM and good agreement in the Z axis between GT9X and wGT3X on the hip placement site, whereas there was good agreement in steps, X axis, Y axis, Z axis, and VM on the wrist placement site.

**Figure 1:**
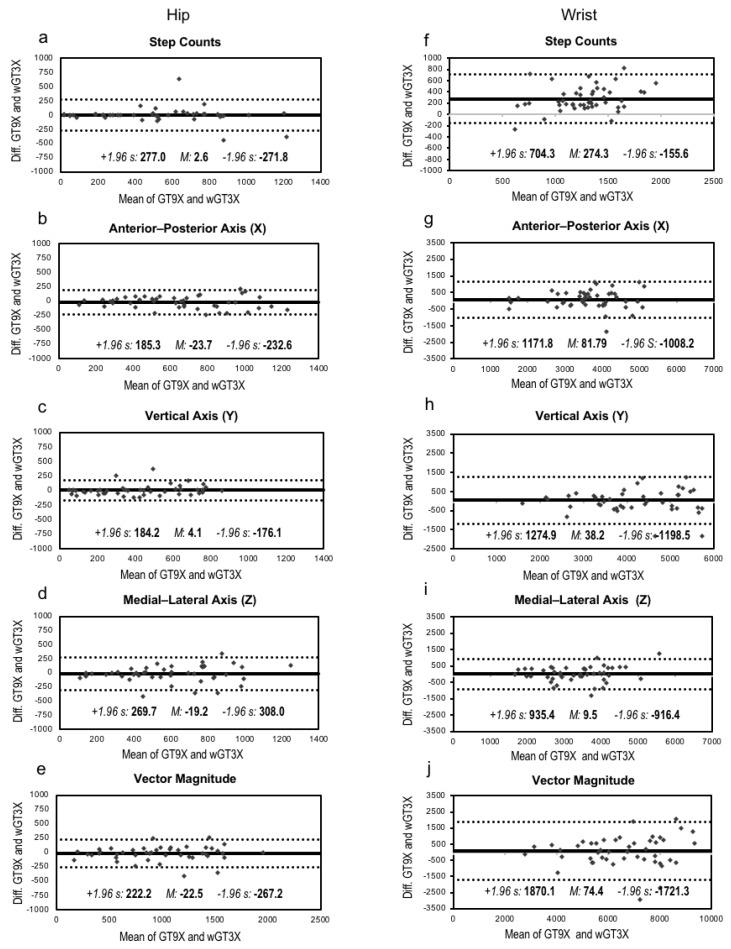
Bland-Altman plots and intraclass correlation (ICC). The solid line represents the mean bias (*M*), whereas the dotted line indicates the limits of agreement computed as the mean bias plus or minus 1.96 times its standard deviation (s). ICC represents intraclass correlation that measures the agreement of measurements between the GT9X and wGT3X. Data in Figure (**a**–**e**) are from the hip-worn monitors, whereas data in Figure (**f**–**j**) are from the wrist-worn monitors.

The Bland-Altman plots illustrating the agreement between GT9X and wGT3X in steps and tri-axis activity counts with means and a 95% confidence interval are depicted in Figure 1. We found considerable agreement between GT9X and wGT3X on the hip site, as indicated by mean bias differences of 1.1% in steps, −4.0% in X axis, −4.4% in Y axis, −7.4% in Z axis, and −4.2% in VM. We found reasonably good agreements on the wrist, as indicated by mean bias differences of 2.1% in X axis, 0.5% in Y axis, 0.1% in Z axis, and 0.9% in VM; however, there was relatively poor agreement in terms of steps (a mean bias difference of 21.4%) between the monitors.

**Figure 2:**
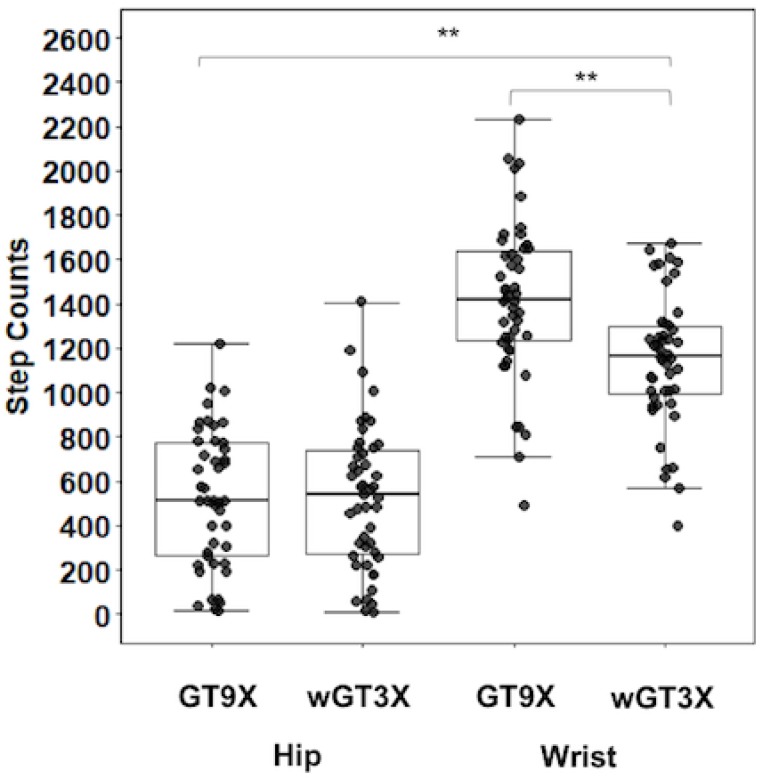
Average step counts. Box plot with scatter represents 25th percentile at bottom and 75th percentile at top with the highest, median, and lowest value. ** *p* < 0.001: higher steps in GT9X vs. wGT3X on the wrist; higher steps in wrist vs. hip.

The step difference between the monitors was significant on the wrist (*F*_1,47_ = 73.42, *p* < 0.001), with steps reported from the GT9X (1418.5 ± 354.1) higher than that from the wGT3X (1144.2 ± 285.9). However, this difference was not significant on the hip (*F*_1,45_ = 0.02, *p* = 0.903), which had similar step counts reported by the GT9X (525.0 ± 310.6) and the wGT3X (522.3 ± 329.3) (Figure 2). There was no significant difference in the X, Y, Z, or VM (CPM) between monitors placed on the wrist and those placed on the hip (Table 2). The wrist-worn monitors produced higher steps (Figure 2), tri-axial counts, and VM than the hip-worn monitors (all, *p* < 0.001) (Table 2).

### 3.3. Time Spent in Sedentary and Physical Activity Intensity Levels

**Figure 3:**
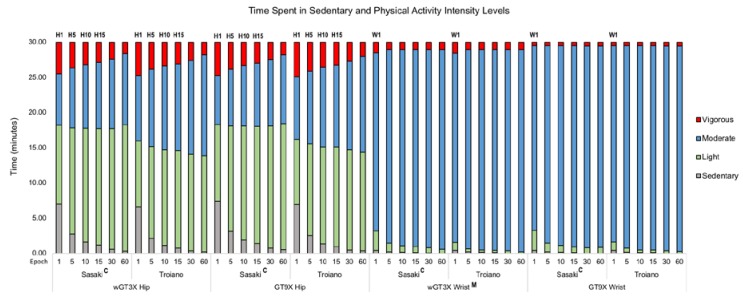
Time spent in sedentary and physical activity intensity levels in epochs. ^H1^ longer sedentary behavior (SB) and shorter light physical activity (LPA) (1 s vs. other longer five epochs: all, *p* < 0.001) and shorter moderate physical activity (MPA) and longer vigorous physical activity (VPA) (1 s vs. 10, 15, 30, and 60 s: all, *p* < 0.001); ^H5^ longer SB (5 s vs.10, 15, 30, and 60 s: all, *p* < 0.001), shorter LPA (5 s vs. 60 s: *p* = 0.039); shorter MPA (5 s vs. 30 and 60 s: *p* = 0.010 and *p* < 0.001, respectively), longer VPA (5 s vs. 30 s and 60 s: all, *p* < 0.001); ^H10^ longer SB (10 s vs. 60 s: *p* = 0.012), shorter MPA (10 s vs. 60 s: *p* = 0.015), longer VPA (10 s vs. 60 s: *p* < 0.001); ^H15^ longer VPA (15 s vs. 60 s, *p* < 0.001); ^W1^ longer SB (1 s vs. 10, 15, 30, and 60 s: *p* = 0.038, *p* = 0.019, *p* <0.001, and *p* < 0.001, respectively), longer LPA, and shorter MPA (1 s vs. other five longer epochs: all, *p* < 0.001); ^C^ longer LPA and shorter MPA (Sasaki vs. Troiano: all epochs, *p* < 0.001); ^M^ longer VPA (wGT3X vs. GT9X, *p* = 0.021). ^H, W, C, and M^ are indicated as hip, wrist, cut-point set, and monitor, respectively, while number is presented as epoch length. Data are presented as means in minutes.

Since there were no significant interactions between epoch lengths, monitors, and activity cut-point sets, we ran an analysis on the effect of epoch lengths on sedentary and physical activity intensity levels assessed using the two monitors and the two sets of activity cut-points (Figure 3 and Supplemental Table S1). The effect of epoch lengths on activity levels was significant on the hip (*F*_5, 1080_ = 6.26, *p* < 0.001), indicating that the shortest epoch (1 s) was significantly related to more time spent in SB (all, *p* < 0.001), less time spent in LPA (all, *p* < 0.001) and in MPA (all, *p* < 0.001) and more time spent in VPA (all, *p* < 0.001) compared to the other five longer epochs. In addition, the effect of epoch lengths on activity levels was significant on the wrist (*F*_5, 1104_ = 3.89, *p* = 0.002), indicating that the shortest epoch was significantly associated with more time spent in SB (all, *p* < 0.001) and in LPA (all, *p* < 0.001) and less time spent in MPA (all, *p* < 0.001) compared to the other five longer epochs. When we categorized physical activity using Sasaki’s activity cut-point set, we found that more time was spent in LPA and a shorter time was spent in MPA on the hip (*F*_1, 1080_ = 32.94, *p* < 0.001) and the wrist (*F*_1, 1104_ = 5.76, *p* = 0.017) compared to our results obtained using Troiano’s activity cut-point set. The wGT3X monitor indicated a longer time spent in VPA compared to the GT9X monitor on the wrist (*F*_1, 1104_ = 4.13, *p* = 0.042).

### 3.4. Sedentary and Physical Activity Levels Between Heart Rate and Activity Counts in Epochs

**Figure 4:**
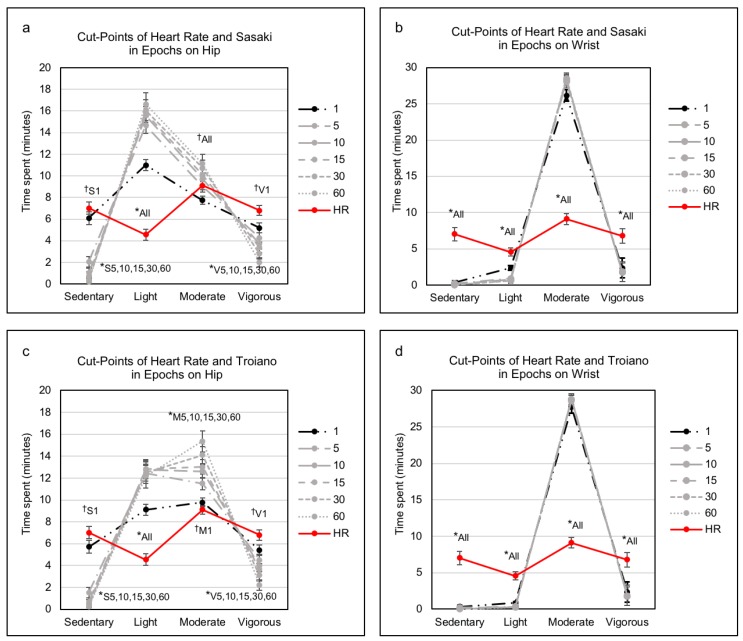
Sedentary and physical activity levels between heart rate and activity counts in epochs. In comparing heart rate, ^†^ indicates a nonsignificant difference (*p* > 0.05) with an epoch, whereas * denotes a significant difference (*p* < 0.001) with epochs. The S, M, and V are indicated as sedentary, moderate, and vigorous, respectively, whereas a number and all are represented as an epoch and all epochs, respectively. HR, heart rate. Data are presented as mean ± standard error in minutes.

As depicted in Figure 4a,c and in Supplemental Table S2, the time spent (min) in sedentary and physical activity intensity levels for the hip placement site, derived from the two cut-point sets of activity counts, was comparable to the indirect assessment of intensity using HR across six epoch lengths. For instance, the HR-derived measure of SB (7.0 ± 5.6 min) was similar to that determined using a 1-s epoch with both the Sasaki cut-point set (5.9 ± 3.6 min; *p* = 0.313) and the Troiano cut-point set (5.7 ± 3.6 min; *p* = 0.159) but differed from that obtained from the longer epoch lengths in either cut-point set (all, *p* < 0.001). The HR-derived measures of LPA (4.6 ± 3.4) were not similar to the intensity level determined using either of the activity cut-point sets across all epoch lengths (all, *p* < 0.001). The HR-derived measures of MPA (9.1 ± 4.7 min) were similar to that determined for all epochs (all, *p* > 0.05) using the Sasaki cut-point set but only for a 1-s epoch (9.8 ± 2.5 min; *p* = 0.345) using the Troiano cut-point set. The HR-derived measures of VPA (6.8 ± 6.3 min) were similar to the activity count intensity measure determined using a 1-s epoch in the Sasaki (5.2 ± 2.8 min; *p* = 0.06) and in the Troiano (5.4 ± 2.9 min; *p* = 0.102) but differed from those determined using the longer epoch lengths (all, *p* < 0.001).

On the wrist placement site (as shown in Figure 4b,d and in Supplemental Table S2), the HR-derived intensity measures of SB, LPA, MPA, and VPA were not comparable to those determined using either cut-point set when compared across all epochs (all, *p* < 0.001, respectively).

Additionally, as shown in Supplemental Table S3, there were similar results in sedentary and various physical activity levels at either placement site using either cut-point set when compared separately with GT9X and wGT3X.

## 4. Discussion

In this study, using an acute bout of exergaming play with two recent generations of ActiGraph monitors, we found that (1) intermonitor differences in steps and activity counts between wGT3X and GT9X were not significant on the hip placement site but were significant in terms of step counts on the wrist placement site; and (2) a 1-s epoch of activity counts obtained from hip-worn activity monitors was the best choice for estimating sedentary and physical activity intensity levels in an exergaming setting when compared with measures of relative exercise intensity using HR. We believe that our work is the first to compare indirect activity intensity measures using HR with activity counts using different epoch lengths, which could be a practical and applicable method for the accuracy of physical activity assessment.

Since newer activity monitor models are continuously being produced by the manufacturers (replacing previous models), researchers have validated outputs (e.g., steps, activity counts) of activity monitors for the accuracy of physical activity assessment. Our results indicated that the differences in steps between wGT3X and GT9X depended on the placement site, although there were strong associations between both monitors on the hip and wrist. More specifically, intermonitor differences for steps between the hip worn-monitors were not significantly different and were generally in good agreement. For these monitors, bias was close to zero, indicating that they were producing similar results, and the 95% limits of agreement were small, suggesting that the hip-worn monitors could be used as an alternative to measure steps. Additionally, there were similar patterns in tri-axial counts, especially on the vertical axis where steps are calculated in the ActiLife step-counting algorithm [1,22].

Our findings are consistent with those of previous studies using other ActiGraph models. These studies showed considerable intermonitor agreement for the vertical axis counts between GT1M and GT3X in young adults during treadmill exercise [4], among GT1M, GT3X, and GT3X+ in children and adolescents with lab-based activities [20], and between the GT3X+ and GT9X in young adults with lab-based activities [21]. However, we observed a relatively poor intermonitor agreement for step counts between the wrist-worn wGT3X and GT9X, as indicated by large and significant intermonitor differences, but reasonably good intermonitor agreement in the vertical and other two axis counts. Some studies have examined possible factors for an intermonitor difference in steps or activity counts. For example, ActiGraph’s low-frequency extension filter (the detection of lower amplitude movements) affects the difference in step or activity counts within different generation models (GT3X+ vs. 7164) [37] or in the same models (GT3X+) [38]. In addition, ActiGraph’s sampling frequency (the processing of raw acceleration data to activity counts) influences the discrepancy in activity counts within the same models (GT3X+) [39]. Since we used the same sampling frequency (30 Hz) and a normal filter instead of low-frequency extension filter when we compared the wGT3X and the GT9X, the source of the discrepancy in steps between the wrist-worn monitors remains unclear.

A recent study [22] compared step outputs between hip and wrist-worn ActiGraph monitors and between wrist-worn GT3X+ and GT9X monitors during treadmill walking and showed that the discrepancy in tri-axial orientations between GT9X and GT3X+ or other previous ActiGraph monitors might significantly impact step-counting accuracy on the wrist. Further, the ActiGraph step-counting algorithm developed for the hip location might not work for the wrist location [7]. Tudor-Locke et al. [15] examined the accuracy of steps on the hip and wrist placement sites using the same ActiGraph GT3X+ monitors and found the hip site outperformed the wrist site at most treadmill speeds, regardless of the bandpass filter. Moreover, we cannot rule out the possibility that the discrepancy in step counts between the wGT3X and GT9X may be due to differences in individual movement patterns. Since the exergaming we studied requires irregular upper body movements, differences in an individual’s arm motion or speed may affect threshold crossing of the acceleration signal, perhaps inducing less step-counting accuracy on the wrist. Additionally, John et al. [22] report that accelerations detected on the wrist were smaller in magnitude than those at the hip during treadmill walking at the same speed, indicating that a wrist-worn monitor would count fewer steps than a hip-worn monitor. However, we found that the wrist-worn GT3X+ monitors resulted in higher steps than hip-worn monitors, which can be explained by the fact that exergaming play involves more arm movements. Thus, when researchers seek to determine the accuracy of step-counting, it is important to take the placement site into consideration. Our result thus suggests that researchers can select either of the two monitors we used here to conduct exergaming research if the devices are placed on the hip.

Of particular importance, we confirmed that epoch lengths differentially influenced assessment of sedentary and different physical activity levels, which is consistent with the previous studies. We found that time spent in SB and physical activity intensity levels varied when assessed using different epoch lengths (1, 5, 10, 15, 30, and 60 s). For instance, as epoch lengths decreased on the hip-worn monitors, estimates of SB and VPA increased while estimates of LPA and MPA decreased. We observed similar patterns in SB and MPA but a different pattern in LPA on the wrist-worn monitors. Our findings here are consistent with those of previous studies showing a varying effect of epoch length with earlier generations of ActiGraph monitors placed on the hip for seven days in a free-living condition. For example, Edwardson and Gorely [26] used single-activity cut-points (i.e., Freedson) and observed that shorter epoch lengths among the four epochs (5, 15, 30, and 60 s) were associated with more time spent in SB and VPA and less time spent in MVPA, MPA, and LPA in children wearing an ActiGraph GT1M monitor on the hip. Banda et al. [25] used multiple activity cut-points (e.g., Evenson, Treuth, Puyan) and showed that shorter epoch lengths among the six epochs (1, 5, 10, 15, 30, and 60 s) were related to more time spent in SB, MPA, and VPA and less time spent in LPA in children wearing the ActiGraph GT3X+ monitor on the hip. Finally, a study examining physical activity levels in middle-aged adults found that an epoch of 4 s among the three epochs (4, 20, and 60 s) was associated with longer time spent in VPA and shorter in LPA [28]. The consistency in findings from the previous studies and our study might be associated with the form and intensity of an intermittent and spontaneous physical activity in which shorter epoch lengths such as a 1-s epoch [25] or a 2-s epoch [29] were the most appropriate epoch length to capture short bouts of vigorous or more intense physical activity. The physical activity form and/or intensity might be comparable to the exergaming play we used, which is characterized by rapid changes from sedentary to intense physical activity occurring frequently in a short period [40]. Taken together, although the results from different epoch lengths vary, shorter epoch lengths may be appropriate for capturing short bouts, especially in more intense physical activity.

However, previous studies examining the effect of epochs on assessment of physical activity levels have not apparently compared relative or absolute measures of exercise intensity [25,26,28], which might attenuate their findings. We compared physical activity intensity based on a cut-point set of HR with each of two cut-point sets of activity counts and found that the amount of time spent in SB, MPA, and VPA with the 1-s epoch length on the hip-worn monitors was similar to that in SB, MPA, and VPA of the HR but that this did not hold for any of the other longer epoch lengths from the hip- or wrist-worn monitors (Figure 4). This is a novel result with respect to previous validations of cut-points of activity counts or raw data (accelerations) against indirect calorimetry (absolute measure) and HR monitoring (relative measure) for physical activity intensity. Previous studies have validated cut-points for physical activity intensity using indirect calorimetry as a gold standard measure of energy expenditure and metabolic equivalent for regular physical activities (e.g., treadmill walking/running) [4,41]. Other studies have used HR monitoring as a relatively less expensive but feasible instrument to support the validation of cut-points with different analytical methods. For instance, Ozemek et al. [42] suggested that activity counts were comparable to heart rate using %HRR at relative moderate (40% HRR) and vigorous (60% HRR) intensities but added that this depended on individual fitness levels. Two studies compared raw data and HR for sedentary activities and different intensity levels of physical activity and showed a strong correlation (*r* = 0.97) [43] and excellent agreement (receiver operating characteristic area under the curve = 0.99) [44]. Thus, measures of indirect calorimetry or HR monitoring can be comparable to intensity assessed using cut-points of activity counts. We found that a 1-s epoch length in conjunction with a hip-worn monitor was the most similar to HR-derived measures and should be the most accurate method for measuring sedentary and various physical activity intensity levels in an exergaming setting.

We should note some important limitations to our results. Based on our statistical methods, we cannot determine the better of the two monitors we tested, but either of the two models of activity monitors can be used interchangeably on the hip placement site. Although hip-worn monitors seemed to be more appropriate for assessing step counts as well as physical activity intensity levels, we cannot generalize our results to other physical activity conditions or other activity monitor brands. Further, even though we demonstrated that a 1-s epoch would be the most appropriate epoch length for detecting short bursts of intense physical activity, it is unclear how an estimate of sedentary and physical activity intensity levels can be comparable to objective measures of energy expenditure [45]. Thus, the fact that we did not use an indirect calorimetry technique as a criterion measure for physical activity intensity [46] may be considered a limitation and therefore may require further investigation.

## 5. Conclusions

We demonstrated that the activity monitors we used are valid and reliable devices for step-counting accuracy when placed on the hip, and that the hip compared to the wrist is also a more appropriate placement site for accurately measuring levels of physical activity intensity. We suggest that a 1-s epoch is the best choice for data processing and analysis of activity counts for the activity monitors we used in the present study for physical activity assessment. We further recommend that heart rate can be used as a comparator for the validation of cut-points from activity counts. Our findings are applicable in other clinical, research, or school settings focusing on intermittent physical activities similar to exergaming.

## Figures and Tables

**Table 1:** Interclass correlation coefficient between GT9X and wGT3X in steps and activity counts.

		Interclass Correlation Coefficient	95% Confidence Interval
Hip	Steps	0.94	0.89–0.97
	Anterior-posterior (X) axis	0.93	0.88–0.96
	Vertical (Y) axis	0.93	0.87–0.96
	Medial-lateral (Z) axis	0.86	0.77–0.92
	Vector magnitude	0.95	0.92–0.97
Wrist	Steps	0.80	0.66–0.88
	Anterior-posterior (X) axis	0.83	0.71–0.90
	Vertical (Y) axis	0.87	0.77–0.92
	Medial-lateral (Z) axis	0.86	0.77–0.92
	Vector magnitude	0.89	0.81–0.94

**Table 2:** Average of activity counts based on the six epoch lengths.

	wGT3X Hip						GT9X Hip					
	1	5	10	15	30	60	1	5	10	15	30	60
Vertical Axis (Y), Counts	6.8	33.9	67.9	101.9	204.0	407.7	7.0	34.8	69.7	104.6	209.2	419.0
	±3.9	±19.3	±38.6	±57.9	±116.1	±232.9	±4.2	±21.0	±42.1	±63.1	±126.4	±253.4
Anterior-Posterior Axis (X), Counts	11.0	55.0	110.1	165.3	330.9	661.9	10.6	53.0	106.0	159.1	318.5	638.0
	±4.9	±24.6	±49.3	±74.0	±148.1	±296.6	±4.8	±23.9	±47.7	±71.6	±143.4	±287.6
Medial-Lateral Axis (Z), Counts	9.9	49.4	98.9	148.4	297.2	594.5	9.6	48.2	96.5	144.8	289.8	580.5
	±4.5	±22.7	±45.5	±68.2	±136.6	±273.3	±4.9	±24.4	±48.8	±73.1	±146.5	±293.5
Vector Magnitude, Counts	18.8	89.2	175.5	261.1	517.3	1026.2	18.5	87.8	172.6	256.9	508.4	1010.6
	±8.0	±38.0	±74.8	±111.5	±221.3	±440.0	±8.3	±39.3	±77.2	±115.2	±228.3	±454.7
	wGT3X Wrist **						GT9X Wrist **					
Vertical Axis (Y), Counts	71.1	355.6	711.4	1067.5	2137.7	4287.1	61.0	305.1	610.4	915.9	1833.7	3675.7
	±19.6	±98.0	±196.1	±294.5	±589.6	±1184.6	±15.9	±79.6	±159.3	±239.1	±478.7	±960.0
Anterior-Posterior Axis (X), Counts	59.6	298.2	596.7	895.4	1793.0	3596.0	71.7	358.8	717.8	1077.1	2156.6	4323.0
	±15.6	±77.9	±155.9	±234.1	±468.9	±941.3	±20.8	±104.3	±208.6	±313.0	±626.4	±1254.6
Medial-Lateral Axis (Z), Counts	55.8	279.0	558.3	837.8	1677.7	3364.4	55.9	279.9	559.9	840.2	1682.3	3372.0
	±14.5	±72.5	±145.1	±218.1	±436.5	±877.3	±15.6	±78.2	±156.4	±234.8	±469.7	±941.1
Vector Magnitude, Counts	115.9	561.1	1113.0	1663.4	3314.3	6625.0	117.2	567.7	1125.9	1682.5	3352.3	6698.1
	±28.2	±137.5	±273.7	±410.3	±819.9	±1645.9	±30.0	±146.5	±291.7	±436.9	±872.7	±1746.1

** *p* < 0.001: higher tri-axial activity counts and vector magnitude in the wrist than the hip.

## Supplementary Materials

The following are available online at http://www.mdpi.com/2077-0383/7/9/268/s1, Figure S1: Orientation of GT9X and wGT3X, Table S1: Time spent on sedentary and physical activity intensity levels in epochs, Table S2: Sedentary and physical activity levels between heart rate and activity counts in epochs with two activity cut-point sets, Table S3: Sedentary and physical activity levels between heart rate and activity counts in epochs with two activity cut-point sets and two activity monitors.
