# Supplementary material for: Application and Validation of Activity Monitors’ Epoch Lengths and Placement Sites for Physical Activity Assessment in Exergaming
Source: J Clin Med. 2018 Sep 11;7(9):268. doi: 10.3390/jcm7090268 (PMC6162850; doi:10.3390/jcm7090268)
Supplement: Supplementary file 1 [file jcm-07-00268-s001.pdf]

Supplementary materials: Application and Validation of Activity Monitors' Epoch Lengths and Placement Sites for Physical Activity Assessment in Exergaming

Table S1. Time spent on sedentary and physical activity intensity levels in epochs.

		wGT3X Hip						GT9X Hip					
		**1**	**5**	**10**	**15**	**30**	**60**	**1**	**5**	**10**	**15**	**30**	**60**
Troiano	Sedentary, min	6.6	2.1	1.1	0.8	0.4	0.2	6.9	2.5	1.3	0.9	0.5	0.3
		±4.5	±3.5	±2.7	±2.6	±1.9	±1.3	±4.7	±3.8	±3.1	±2.8	±2.1	±1.6
	Light, min	9.4	13.0	13.6	13.8	13.7	13.6	9.3	13.1	13.8	14.2	14.2	14.0
		±3.1	±4.7	±5.7	±6.2	±7.0	±7.5	±3.1	±4.9	±5.8	±6.5	±7.1	±8.2
	Moderate, min	9.3	11.0	11.9	12.3	13.3	14.4	8.9	10.3	11.4	11.6	12.6	13.7
		±3.5	±4.6	±5.1	±5.4	±5.9	±6.7	±3.2	±4.2	±4.9	±5.2	±6.0	±7.2
	Vigorous, min	4.7	3.9	3.4	3.1	2.6	1.8	4.9	4.1	3.6	3.3	2.7	2.0
		±3.3	±3.3	±3.2	±3.3	±3.1	±2.8	±3.2	±3.3	±3.3	±3.2	±3.2	±2.9
Sasaki	Sedentary, min	7.0	2.7	1.6	1.2	0.6	0.3	7.4	3.1	1.9	1.4	0.7	0.5
		±4.6	±3.9	±3.3	±2.9	±2.5	±1.6	±4.8	±4.2	±3.8	±3.4	±2.8	±2.3
	Light, min	11.2	15.1	16.2	16.5	17.1	17.9	10.9	15.0	16.2	16.7	17.4	17.9
		±3.2	±4.6	±5.2	±5.6	±6.5	±7.0	±3.3	±4.9	±5.4	±6.0	±6.8	±7.5
	Moderate, min	7.3	8.5	9.0	9.4	9.9	10.1	7.0	8.1	8.6	9.0	9.4	9.8
		±3.0	±3.9	±4.3	±4.7	±5.2	±5.9	±2.9	±3.9	±4.3	±4.7	±5.5	±6.5
	Vigorous, min	4.5	3.6	3.2	2.9	2.4	1.6	4.7	3.8	3.3	3.0	2.4	1.8
		±3.1	±3.2	±3.1	±3.1	±2.9	±2.7	±3.1	±3.1	±3.1	±3.1	±3.0	±2.8

		wGT3X Wrist						GT9X Wrist					
		**1**	**5**	**10**	**15**	**30**	**60**	**1**	**5**	**10**	**15**	**30**	**60**
Troiano	Sedentary, min	0.4	0.2	0.1	0.1	0.0	0.0	0.4	0.2	0.1	0.1	0.0	0.0
		±1.8	±1.2	±0.9	±0.7	±0.0	±0.0	±1.7	±1.1	±0.7	±0.5	±0.0	±0.0
	Light, min	1.2	0.5	0.3	0.3	0.3	0.2	1.2	0.6	0.3	0.4	0.4	0.2
		±1.5	±1.2	±1.1	±1.1	±1.3	±1.2	±1.8	±1.3	±1.1	±1.2	±1.4	±0.9
	Moderate, min	26.9	28.3	28.5	28.6	28.6	28.7	27.9	28.8	29.1	29.0	29.1	29.2
		±6.5	±5.4	±5.3	±5.2	±5.3	±5.2	±4.4	±3.9	±3.7	±3.8	±3.8	±4.0
	Vigorous, min	1.6	1.0	1.0	1.0	1.1	1.1	0.5	0.5	0.5	0.5	0.5	0.6
		±6.1	±4.9	±5.0	±5.0	±5.1	±5.1	±3.5	±3.5	±3.5	±3.5	±3.6	±3.9
Sasaki	Sedentary, min	0.4	0.2	0.1	0.1	0.0	0.0	0.4	0.2	0.1	0.1	0.0	0.0
		±1.8	±1.2	±0.9	±0.7	±0.1	±0.0	±1.7	±1.1	±0.8	±0.6	±0.0	±0.0
	Light, min	2.8	1.3	0.9	0.8	0.8	0.6	2.9	1.3	1.0	0.8	0.8	0.9
		±2.4	±1.9	±1.8	±1.8	±2.1	±2.2	±2.5	±2.2	±1.9	±1.9	±1.9	±2.0
	Moderate, min	25.3	27.5	27.9	28.0	28.2	28.3	26.2	28.0	28.4	28.6	28.7	28.6
		±6.5	±5.6	±5.5	±5.5	±5.6	±5.5	±4.6	±4.3	±4.0	±4.1	±4.0	±4.2
	Vigorous, min	1.5	1.0	1.0	1.0	1.1	1.1	0.5	0.5	0.5	0.5	0.5	0.6
		±6.0	±4.9	±5.0	±5.0	±5.1	±5.1	±3.4	±3.5	±3.5	±3.5	±3.6	±3.8

Table S2. Sedentary and physical activity levels between heart rate and activity counts in epochs with two activity cut-point sets.

	Epoch	Hip								Wrist							
		Sedentary		Light		Moderate		Vigorous		Sedentary		Light		Moderate		Vigorous	
HR		7.0	±5.6	4.6	±3.4	9.1	±4.7	6.8	±6.3	7.0	±5.6	4.6	±3.4	9.11	±4.7	6.8	±6.3
Sasaki	1	6.1	±3.7 †	11.0	±3.3 *	7.7	±2.3 †	5.2	±2.8†	0.3	±1.7 *	2.3	±2.0 *	26.2	±5.1 *	2.3	±8.9 *
	5	2.1	±3.1 *	14.7	±4.7 *	9.0	±3.2 †	4.3	±3.0 *	0.2	±1.3 *	0.9	±1.5 *	28.0	±4.8 *	1.8	±8.2 *
	10	1.0	±2.7 *	15.6	±5.0 *	9.7	±3.5 †	3.7	±3.0 *	0.1	±0.9 *	0.7	±1.5 *	28.3	±4.6 *	1.8	±8.2 *
	15	0.7	±2.4 *	15.8	±5.4 *	10.1	±3.9 †	3.4	±3.0 *	0.1	±0.7 *	0.6	±1.6 *	28.4	±4.7 *	1.8	±8.3 *
	30	0.4	±2.0 *	16.1	±5.9 *	10.7	±4.6 †	2.8	±2.9 *	0.0	±0.0 *	0.6	±1.9 *	28.5	±4.7 *	1.8	±8.6 *
	60	0.2	±1.5 *	16.6	±6.6 *	11.1	±5.6 †	2.0	±2.8 *	0.0	±0.0 *	0.6	±2.0 *	28.5	±4.8 *	1.9	±8.8 *
Troiano	1	5.7	±3.6 †	9.1	±3.2 *	9.8	±2.5 †	5.4	±2.9 †	0.3	±1.7 *	0.8	±1.2 *	27.7	±5.0 *	2.4	±8.9 *
	5	1.5	±3.0 *	12.4	±4.6 *	11.5	±3.5 *	4.5	±3.1 *	0.2	±1.2 *	0.3	±0.8 *	28.6	±4.5 *	1.8	±8.3 *
	10	0.7	±2.4 *	12.7	±5.2 *	12.6	±3.9 *	4.0	±3.1 *	0.1	±0.8 *	0.2	±0.9 *	28.7	±4.4 *	1.8	±8.3 *
	15	0.5	±2.3 *	12.8	±5.5 *	13.0	±4.2 *	3.7	±3.2 *	0.1	±0.7 *	0.3	±0.9 *	28.8	±4.4 *	1.8	±8.3 *
	30	0.3	±1.9 *	12.5	±6.0 *	14.1	±4.9 *	3.1	±3.1 *	0.0	±0.0 *	0.3	±1.3 *	28.8	±4.5 *	1.8	±8.6 *
	60	0.2	±1.4 *	12.2	±6.8 *	15.4	±6.0 *	2.2	±2.9 *	0.0	±0.0 *	0.2	±1.0 *	28.8	±4.5 *	1.9	±8.9 *

† indicates a non-significant difference ($P > 0.05$) between an epoch and the HR. whereas * denotes a significant difference ($P < 0.001$) between an epoch with the HR.

Table S3. Sedentary and physical activity levels between heart rate and activity counts in epochs with two activity cut-point sets and two activity monitors.

		Epoch	Hip								Wrist							
			Sedentary		Light		Moderate		Vigorous		Sedentary		Light		Moderate		Vigorous	
	HR		7.0	±5.6	4.6	±3.4	9.1	±4.7	6.8	±6.3	7.0	±5.6	4.6	±3.4	9.11	±4.7	6.8	±6.3
Sasaki	wGT3X	1	5.8	±3.6 †	11.2	±3.4 *	8.0	±2.5 †	5.0	±3.1 *	0.3	±1.8 *	2.3	±2.2 *	25.6	±6.8 *	1.8	±6.4 *
		5	1.8	±3.0 *	14.8	±4.6 *	9.4	±3.4 †	4.0	±3.2 *	0.2	±1.3 *	1.0	±1.7 *	27.7	±5.9 *	1.2	±5.3 *
		10	0.9	±2.5 *	15.6	±5.0 *	10.0	±3.7 †	3.5	±3.1 *	0.2	±1.0 *	0.8	±1.6 *	27.9	±5.8 *	1.2	±5.3 *
		15	0.6	±2.4 *	15.7	±5.3 *	10.4	±4.0 †	3.2	±3.2 *	0.1	±0.8 *	0.7	±1.7 *	28.0	±5.8 *	1.2	±5.3 *
		30	0.4	±2.1 *	16.0	±5.9 *	11.0	±4.6 *	2.7	±3.0 *	0.0	±0.1 *	0.7	±2.1 *	28.1	±5.9 *	1.2	±5.4 *
		60	0.2	±1.6 *	16.6	±6.5 *	11.3	±5.4 *	1.9	±2.9 *	0.0	±0.0 *	0.5	±2.2 *	28.32	±5.9 *	1.2	±5.5 *
	GT9X	1	6.2	±3.9 †	10.9	±3.4 *	7.6	±2.5 †	5.3	±2.9 †	0.3	±1.7 *	2.3	±2.0 *	26.8	±4.6 *	0.6	±3.7 *
		5	2.2	±3.3 *	14.6	±4.8 *	8.9	±3.4 †	4.3	±3.1 *	0.2	±1.2 *	0.8	±1.7 *	28.4	±4.4 *	0.6	±3.7 *
		10	1.1	±2.8 *	15.6	±5.1 *	9.6	±3.7 †	3.7	±3.1 *	0.1	±0.8 *	0.7	±1.7 *	28.6	±4.2 *	0.6	±3.7 *
		15	0.8	±2.5 *	15.8	±5.6 *	9.9	±4.1 †	3.4	±3.2 *	0.1	±0.7 *	0.6	±1.7 *	28.8	±4.3 *	0.6	±3.8 *
		30	0.4	±2.0 *	16.2	±6.1 *	10.6	±4.9 †	2.8	±3.1 *	0.0	±0.0 *	0.6	±1.8 *	28.8	±4.2 *	0.6	±3.9 *
		60	0.2	±1.4 *	16.6	±7.0 *	11.1	±6.0 †	2.1	±2.9 *	0.0	±0.0 *	0.7	±2.0 *	28.7	±4.4 *	0.6	±4.1 *
Troiano	wGT3X	1	5.5	±3.5 †	9.2	±3.2 *	10.1	±2.9 †	5.2	±3.2 *	0.3	±1.7 *	0.9	±1.3 *	27.0	±6.9 *	1.8	±6.5 *

	5	1.3	±2.8 *	12.4	±4.6 *	12.0	±3.9 *	4.3	±3.3 *	0.2	±1.3 *	0.3	±0.8 *	28.3	±5.7 *	1.2	±5.3 *
	10	0.6	±2.2 *	12.6	±5.2 *	13.0	±4.3 *	3.8	±3.3 *	0.1	±0.9 *	0.2	±0.9 *	28.4	±5.6 *	1.2	±5.4 *
	15	0.4	±2.2 *	12.6	±5.5 *	13.5	±4.5 *	3.4	±3.4 *	0.1	±0.8 *	0.2	±0.9 *	28.5	±5.5 *	1.2	±5.3 *
	30	0.3	±1.9 *	12.2	±6.0 *	14.5	±5.1 *	3.0	±3.2 *	0.0	±0.0 *	0.3	±1.3 *	28.5	±5.7 *	1.2	±5.4 *
	60	0.2	±1.4 *	12.0	±6.5 *	15.7	±5.9 *	2.0	±2.9 *	0.0	±0.0 *	0.2	±1.2 *	28.6	±5.5 *	1.2	±5.5 *
GT9X	1	5.9	±3.8 †	9.0	±3.2 *	9.6	±2.7 †	5.5	±3.0 †	0.3	±1.7 *	0.8	±1.3 *	28.3	±4.4 *	0.6	±3.7 *
	5	1.7	±3.1 *	12.4	±4.7 *	11.3	±3.5 *	4.6	±3.2 *	0.2	±1.1 *	0.3	±1.0 *	28.9	±4.1 *	0.6	±3.8 *
	10	0.8	±2.5 *	12.8	±5.3 *	12.4	±4.1 *	4.0	±3.3 *	0.1	±0.7 *	0.3	±1.0 *	29.0	±4.0 *	0.6	±3.7 *
	15	0.6	±2.3 *	13.0	±5.8 *	12.8	±4.4 *	3.7	±3.2 *	0.1	±0.6 *	0.3	±1.1 *	29.0	±4.0 *	0.6	±3.8 *
	30	0.3	±1.8 *	12.7	±6.3 *	13.9	±5.2 *	3.1	±3.3 *	0.0	±0.0 *	0.4	±1.4 *	29.0	±4.1 *	0.6	±3.9 *
	60	0.2	±1.4 *	12.3	±7.3 *	15.2	±6.4 *	2.3	±3.0 *	0.0	±0.0 *	0.2	±0.9 *	29.1	±4.3 *	0.7	±4.2 *

† indicates a non-significant difference ($P > 0.05$) between an epoch and the HR. Whereas * denotes a significant difference ($P < 0.001$) between an epoch with the HR.

Figure S1. Orientation of GT9X and wGT3X.

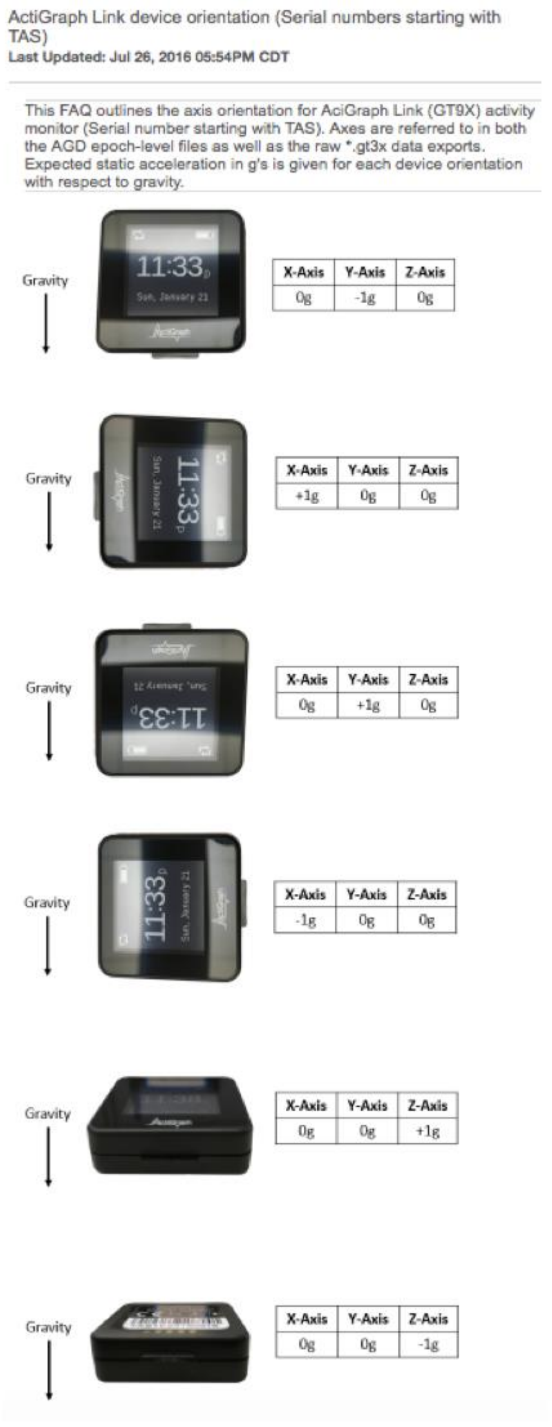

ActiGraph Link device orientation (Serial numbers starting with TAS)

Last Updated: Jul 26, 2016 05:54PM CDT

This FAQ outlines the axis orientation for AciGraph Link (GT9X) activity monitor (Serial number starting with TAS). Axes are referred to in both the AGD epoch-level files as well as the raw *.gt3x data exports. Expected static acceleration in g's is given for each device orientation with respect to gravity.

Gravity

X-Axis	Y-Axis	Z-Axis
0g	-1g	0g

Gravity

X-Axis	Y-Axis	Z-Axis
+1g	0g	0g

Gravity

X-Axis	Y-Axis	Z-Axis
0g	+1g	0g

Gravity

X-Axis	Y-Axis	Z-Axis
-1g	0g	0g

Gravity

X-Axis	Y-Axis	Z-Axis
0g	0g	+1g

Gravity

X-Axis	Y-Axis	Z-Axis
0g	0g	-1g

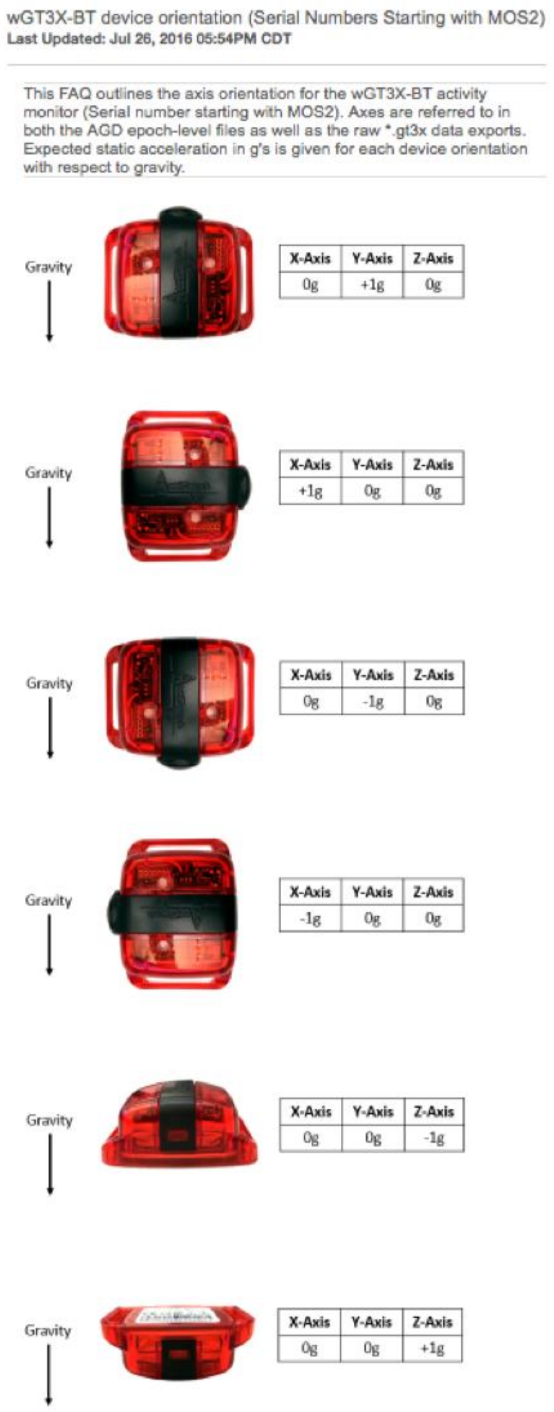

wGT3X-BT device orientation (Serial Numbers Starting with MOS2)

Last Updated: Jul 26, 2016 05:54PM CDT

This FAQ outlines the axis orientation for the wGT3X-BT activity monitor (Serial number starting with MOS2). Axes are referred to in both the AGD epoch-level files as well as the raw *.gt3x data exports. Expected static acceleration in g's is given for each device orientation with respect to gravity.

Gravity

X-Axis	Y-Axis	Z-Axis
0g	+1g	0g

Gravity

X-Axis	Y-Axis	Z-Axis
+1g	0g	0g

Gravity

X-Axis	Y-Axis	Z-Axis
0g	-1g	0g

Gravity

X-Axis	Y-Axis	Z-Axis
-1g	0g	0g

Gravity

X-Axis	Y-Axis	Z-Axis
0g	0g	-1g

Gravity

X-Axis	Y-Axis	Z-Axis
0g	0g	+1g
